# Hypermutation of specific genomic loci of *Pseudomonas putida* for continuous evolution of target genes

**DOI:** 10.1111/1751-7915.14098

**Published:** 2022-06-13

**Authors:** Elena Velázquez, Beatriz Álvarez, Luis Ángel Fernández, Víctor de Lorenzo

**Affiliations:** ^1^ Systems Biology Department Centro Nacional de Biotecnología (CNB‐CSIC) 28049 Madrid Spain; ^2^ Microbiology Department Centro Nacional de Biotecnología (CNB‐CSIC) 28049 Madrid Spain

## Abstract

The ability of T7 RNA polymerase (RNAP^T7^) fusions to cytosine deaminases (CdA) for entering C➔T changes in any DNA segment downstream of a T7 promoter was exploited for hyperdiversification of defined genomic portions of *Pseudomonas putida* KT2440. To this end, test strains were constructed in which the chromosomally encoded *pyrF* gene (the prokaryotic homologue of yeast URA3) was flanked by T7 transcription initiation and termination signals and also carried plasmids expressing constitutively either high‐activity (lamprey's) or low‐activity (rat's) CdA‐RNAP^T7^ fusions. The DNA segment‐specific mutagenic action of these fusions was then tested in strains lacking or not uracil‐DNA glycosylase (UDG), that is *∆ung*/*ung* ^*+*^ variants. The resulting diversification was measured by counting single nucleotide changes in clones resistant to 5‐fluoroorotic acid (5FOA), which otherwise is transformed by wild‐type PyrF into a toxic compound. Although the absence of UDG dramatically increased mutagenic rates with both CdA‐RNAP^T7^ fusions, the most active variant – pmCDA1 – caused extensive appearance of 5FOA‐resistant colonies in the wild‐type strain not limited to C➔T but including also a range of other changes. Furthermore, the presence/absence of UDG activity swapped cytosine deamination preference between DNA strands. These qualities provided the basis of a robust system for continuous evolution of preset genomic portions of *P. putida* and beyond.

## Graphical abstract

This work reports adaptation to *Pseudomonas putida* of a genetic diversification system based on fusions of the T7 RNA polymerase to cytosine deaminases. This platform achieves continuous mutagenesis of defined genomic segments in living cells, enabling evolution of genes of interest.

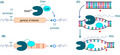

## Introduction

Generation of genotypic and phenotypic variability in bacteria typically involves adoption of *in vivo* DNA variation methods which – in contrast to other schemes – enable mutation, expression and selection steps to be run as a continuous process (Simon *et al*., 2019). Whether occurring spontaneously or stimulated by exogenous agents (Miller *et al*., 1977; Brouwer *et al*., 1981; Akkaya *et al*., 2018) or endogenous mutation triggers (Muteeb and Sen, 2010), modifications of the DNA habitually occur throughout the whole genome of the organism at stake. While this can be beneficial for evolving complex phenotypes (Ibarra *et al*., 2002; Klein‐Marcuschamer *et al*., 2009; Liu *et al*., 2020), most haphazard mutations in a bacterial genome are either neutral or plainly detrimental. Instead, in order to focus *in vivo* diversification into a limited number of sites/genes of the genome, special methods need to be implemented. A successful one is that named MAGE (multiple automated genome engineering) which is based on the Red‐recombination system of lambda phage and the delivery of mutagenic ssDNA oligonucleotides to *Escherichia coli* cells (Wang *et al*., 2009; Isaacs *et al*., 2011; Ronda *et al*., 2016). This system has been later adapted for segment‐specific protein diversification (Nyerges *et al*., 2018; Al‐Ramahi *et al*., 2021) and applied to a suite of non‐*E*. *coli* bacteria thereof (van Kessel and Hatfull, 2007; van Pijkeren and Britton, 2012; Aparicio *et al*., 2016). MAGE and related methods rely on recurrent cycles of ssDNA transformation for raising a large enough library of variants, and they thus still depend on user's action for bringing about the desired site‐specific diversification. MAGE alternatives include adoption of *E*. *coli* strains expressing an error‐prone DNA polymerase I which enables continuous mutagenesis of genes cloned in plasmid vectors with a ColE1‐type origin of replication (Camps *et al*., 2003). Conceptually, similar approaches using other genetic devices have also been successfully implemented in eukaryotic cells (Crook *et al*., 2016; Arzumanyan *et al*., 2018; Ravikumar *et al*., 2018). However, strategies for all *in vivo* continuous evolution of defined segments of bacterial genomes remained parted until the discovery that fusions of cytosine deaminases (CdA) to the bacteriophage T7 RNAP (RNAP^T7^) caused high mutagenic rates C➔T (and accordingly G➔A) in any DNA sequence located downstream of a T7 promoter (Moore *et al*., 2018; Alvarez *et al*., 2020; Chen *et al*., 2020; Cravens *et al*., 2021; Park and Kim, 2021). On this basis, a number of genetic platforms exploiting different base editors fused to RNAP^T7^ have been developed in which the bases of the ssDNA that become exposed along transcription become the substrate of the deamination reaction (Molina *et al*., 2022). This occurrence has been exploited to set off evolution of delimited DNA segments *in vivo* without affecting the rest of the genome. Given the imperfect efficacy of T7 phage transcription terminators (T7_T_) to make RNAP^T7^ to come off the engaged DNA (Moore *et al*., 2018; Tarnowski and Gorochowski, 2022), one useful strategy to define the downstream limit of the mutagenized region involves expression of gRNA‐dCas9 complexes for blocking the progression of the polymerase (Alvarez *et al*., 2020). While the available wealth of results accredits the mutagenic activity of CdA‐RNAP^T7^ fusions in *E. coli*, whether same is applicable to other bacterial species and strains of biotechnological interest remains unknown.

In this work, we have investigated the applicability of such a diversity‐generating device to the Gram‐negative bacterium and metabolic engineering platform *Pseudomonas putida* KT2440 (Nelson *et al*., 2002; Bitzenhofer *et al*., 2021). This strain is a pWW0 plasmid‐free derivative of the soil isolate *P. putida* mt‐2 (Worsey and Williams, 1975), which naturally holds a suite of qualities that make it an adequate host for running strong redox and harsh reactions with a value for industrial and environmental biotechnology (Nikel *et al*., 2014; Ankenbauer *et al*., 2020; Weimer *et al*., 2020). One important characteristic in this respect is the abundance of genes encoding oxido‐reductases able to generate high NAD(P)H, a metabolic trait that facilitates growth in a wide range of aromatic substrates (dos Santos *et al*., 2004; Kim and Park, 2014) and endows a superior tolerance to different types of stress. Owing to this, the robust redox metabolism of *P. putida* KT2440 facilitates heterologous expression of biochemical routes that are hardly supported by other bacteria (Zobel *et al*., 2017). On a practical side, *P. putida* KT2440 is certified to be a safe and non‐pathogenic bacterium for recombinant DNA experiments (Kampers *et al*., 2019). Finally, the number of molecular tools available for all types of genetic manipulations in this bacterium is comparable – and in some cases exceeding – to those existing for *E. coli* (Martin‐Pascual *et al*., 2021). Having such desirable properties, it is no surprise that *P. putida* KT2440 and its derivatives have become chassis of choice for a wide range of metabolic engineering undertakings (Martinez‐Garcia *et al*., 2014; Dvorak and de Lorenzo, 2018; Sanchez‐Pascuala *et al*., 2019; Martinez‐Garcia *et al*., 2020).

The work below documents the ability of CdA‐RNAP^T7^ fusions to boost DNA variability in a preset genomic segment of a *P. putida* KT2440 derivative delimited by a T7 promoter and a T7 terminator, the efficacy and types of mutations caused under various conditions, and the confinement of the diversification regime to the desired DNA portion of the cell's genetic complement. The results not only verify the performance of the approach in this bacterium, but also provide a complete genetic platform for continuous evolution of specified sectors of the *P. putida* KT2440 chromosome.

## Results and discussion

### Rationale for benchmarking activity of CdA‐RNAP^T7^ fusions *in vivo*

**Fig. 1:**
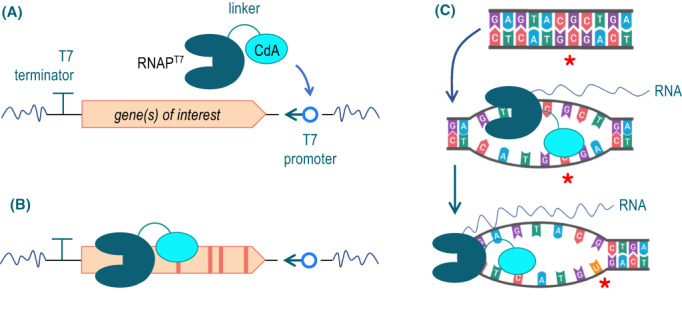
Schematic representation of the segment‐specific mutagenic regime caused by CdA‐RNAP^T7^ fusions *in vivo*. A. The basic arrangement involves one or more gene of interest –or any DNA sequence thereof – flanked by a T7 promoter and a T7 terminator. In the sketch, the direction of transcription from *P*_*T7*_ is opposite to native gene reading, but can be otherwise if desired. The CdA‐RNAP^T7^ fusion is then expressed in trans, bound to *P*_*T7*_ and transcription initiated by the polymerase activity of the fusion. B. As CdA‐RNAP^T7^ proceeds downstream of the *P*_*T7*_, the cytosine deamination moiety may find C residues and change them to U counterparts. C. Mutation occurs as the ssDNA of the transcription bubble exposes C bases, which become substrates of CdA. If the resulting U is not corrected by the repair machinery of the cell, it will base pair with A during the replication process, generating a change from the original C:G pair to a mutated T:A pair. Note that changes can occur, albeit with different frequencies, in the two strands of the DNA. [Colour figure can be viewed at wileyonlinelibrary.com]

**Fig. 2:**
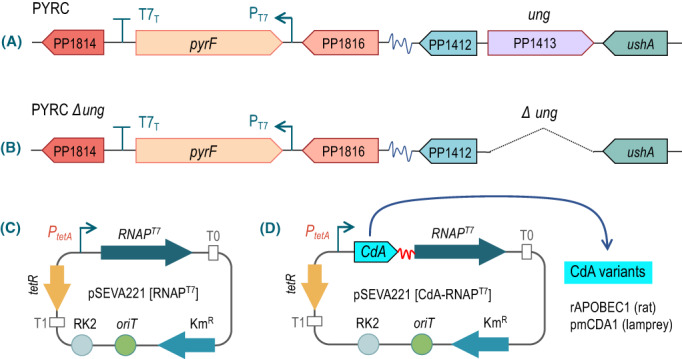
Genetic parts and devices for monitoring action of CdA‐RNAP^T7^ fusions on the *pyrF* gene of *P*. *putida*. A. Organization of the genomic regions of interest in test strain *P*. *putida* PYRC. B. Same, *P*. *putida* PYRC *∆ung*. C. Organization of pSEVA221 [RNAP^T7^] alone. The plasmid is endowed with a low‐copy number origin of replication *oriV* (RK2), an *oriT* to mediate conjugation, a Km^R^ gene and the TetR/*P*_*tetA*_ repressor/promoter pair for transcription of downstream genes. D. Expression plasmids of the two fusion proteins used in this work: rAPOBEC1‐RNAP^T7^ and pmCDA1‐RNAP^T7^. Each cytosine deaminase was fused to the *N*‐terminus domain of RNAP^T7^ through a 28 aa flexible linker (84 nt). [Colour figure can be viewed at wileyonlinelibrary.com]

In order to implement the DNA segment‐targeted mutagenic device shown in Fig. 1 in *P. putida* and – if effective – parameterize its performance, we started by tailoring dedicated strains for reporting important characteristics of the method. The starting isolate was not strain KT2440 but the genome‐edited variant *P. putida* EM42 (Martinez‐Garcia *et al*., 2014; Table S2) that is deleted of the flagellar machinery, which results in higher endogenous levels of NAD(P)H. Furthermore, *P. putida* EM42 lacks a number of instability determinants, that is the four prophages and the Tn7‐like transposon otherwise encoded in the wild‐type genome. The 702 bp genomic segment bearing the *pyrF* gene (PP1815, genomic coordinates 2 040 625–2 041 326 in *P. putida* KT2440; www.pseudomonas.com) was then edited as shown in Fig. S1 to place [i] a T7 promoter (*P*_*T7*_) downstream of the *pyrF* oriented opposite to the course of native transcription and [ii] a T7 terminator (T7_T_) upstream of the innate promoter of the gene (Fig. 2A). *pyrF* encodes the essential enzyme orotidine‐5′‐phosphate decarboxylase, which is the prokaryotic counterpart of yeast URA3 (Galvao and de Lorenzo, 2005). The presence of the enzyme can be selected both positively (growth in minimal medium without uracil) and negatively, as it converts 5‐fluoroorotic acid (5FOA) into 5‐fluorouracil, a toxic compound (Boeke *et al*., 1984). The resulting test strain was called *P. putida* PYRC (Fig. 2A). One further derivative was then constructed (Fig. 2B) by deleting the *ung* gene as described in Experimental Procedures. This gene encodes uracil‐DNA glycosylase (UDG), the enzyme that initiates base repair when U residues accidentally occur in the chromosome (Lindahl *et al*., 1977; Parikh *et al*., 1998; Schormann *et al*., 2014). Since cytosine deaminases convert C to U, *ung* is a key determinant of the mutagenic efficiency of these enzymes (Di Noia and Neuberger, 2002; Rada *et al*., 2002). The growth in minimal media, 5FOA sensitivity and phenotypic characteristics of the resulting strains such as colony size (*P. putida* PYRC and *P. putida* PYRC *∆ung*) were indistinguishable from those of *P. putida* EM42, indicating that the genomic edits had no influence in the physiology of the strains (Fig. S2).

In order to assess the mutagenic action of CdA‐RNAP^T7^ fusions on the thereby arranged *pyrF* sequence, strains *P. putida* PYRC and *P. putida* PYRC *∆ung* were separately transformed with plasmids expressing two different cytosine deaminases fused to the N‐terminus of RNAP^T7^ through a flexible linker (G_3_S)_7_ (Alvarez *et al*., 2020). Specifically, the CdA moieties of the hybrid proteins were those of rat (rAPOBEC1, low‐activity) and lamprey (pmCDA1, high‐activity). These were borne by pSEVA221 [rAPOBEC1‐RNAP^T7^] and pSEVA221 [pmCDA1‐RNAP^T7^], which are described in (Alvarez *et al*., 2020) and sketched in Fig. 2D. Note that the cargoes inserted in the low‐copy number Km^R^ vector pSEVA221 consist of DNA segments for expression of each of the fusions under the control of a *tetR/P*_*tetA*_ system (Bertram and Hillen, 2007). As we pursued a constant supply of the mutagenic source *in vivo*, we preemptively inspected the behaviour of the transcriptional device in *P. putida* by means of a *gfp* reporter gene, which fortunately turned out to be constitutive (Fig. S3). Finally, strains *P. putida* PYRC and *P. putida* PYRC *∆ung* were transformed also with empty vector pSEVA221 as well as with control pSEVA221 [RNAP^T7^], which expressed the RNAP^T7^ under the control of the same *tetR/P*_*tetA*_ as in the other plasmids (Fig. 2C). These constructs provided a reference for diagnosing merely spontaneous mutations in the first case and the sheer effect of counter‐transcribing *pyrF* DNA with an intact RNAP^T7^ in the second. The strain collection resulting in these procedures shaped the genetic platform on which the site‐focused diversity‐generating device of Fig. 1 was tested in *P. putida* as referred to below.

### Setting the baseline for CdA‐unrelated mutagenesis of *pyrF*

**Fig. 3:**
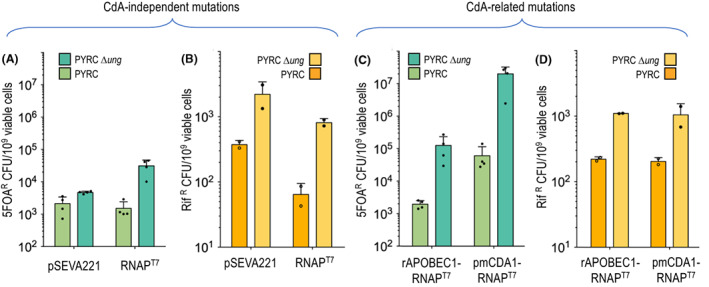
Frequency of *P*. *putida* clones resistant to either 5FOA (putatively in *pyrF*) or rifampicin (putatively in *rpoB*). A. 5FOA^R^ mutants of wild‐type strain *P*. *putida* PYRC and its ∆*ung* derivative transformed with either empty pSEVA221 vector or expressing RNAP^T7^. B. Rif ^R^ mutants of the same strains. Note very different scales of the *Y* axis. C. 5FOA^R^ mutants of *P*. *putida* PYRC and *P*. *putida* PYRC *∆ung* transformed with plasmids expressing fusions of CdA variants rAPOBEC1 and pmCDA1 fused to RNAP^T7^. D. Rif^R^ mutants of the same strains. The mean (bars), standard deviation (lines) and single values (dots) of four (5FOA^R^) or two (Rif^R^) independent biological replicates are shown in each case. [Colour figure can be viewed at wileyonlinelibrary.com]

In order to distinguish the onset of 5FOA^R^ clones due to the genuine action of CdAs from those arising from the inborn variability frequency of *P. putida* and/or high transcription levels from an active T7 promoter, we first focused on strains *P. putida* PYRC and *P. putida* PYRC *∆ung* transformed respectively with control plasmids pSEVA221 and pSEVA221 [RNAP^T7^]. These were plated on M9‐citrate media supplemented with uracil (to assess viability) and M9‐citrate supplemented with uracil and 5FOA (to evaluate *pyrF*‐related mutation frequencies). The same strains were also plated on LB supplemented with rifampicin (Rif) for measuring mutation frequencies of the off‐site gene *rpoB* (Campbell *et al*., 2001; Jatsenko *et al*., 2010). As shown in Fig. 3A, the wild‐type strain with the insert‐free vector pSEVA221 presented mutant frequencies 2.1 × 10^3^ 5FOA^R^ CFU per 10^9^ viable cells of the *P. putida* test strain (Galvao and de Lorenzo, 2005; Aparicio *et al*., 2018). This occurrence increased by ~ two‐fold in the PYRC ∆*ung* strain (4.7 × 10^3^ 5FOA^R^ CFU; Fig. 3A). This was not unexpected, as UDG is part of the general DNA repair machinery and such a moderate rise of basal mutation levels in ∆*ung* mutants has been observed in *P. putida* (Algar *et al*., 2020). These figures thus set the extent of naturally occurring – and somehow anticipated –numbers for the reporter system. Expression of RNAP^T7^‐alone in *P. putida* PYRC and *P. putida* PYRC *∆ung* delivered, however, a different scenario (Fig. 3A). While the wild‐type strain gave frequencies of appearance of 5FOA^R^ clones in the range of those observed in the strains with the empty vector (1.5 × 10^3^ 5FOA^R^ CFU), the same increased by > 10‐fold in the PYRC *∆ung* host (3.1 × 10^4^ 5FOA^R^ CFU). This was not necessarily disadvantageous, but how could it happen? It is known that NH_2_‐containing bases of the ssDNA transiently formed during transcription become highly vulnerable to spontaneous hydrolytic deamination (Lindahl and Nyberg, 1974; Shen *et al*., 1994). Given the high transcriptional activity of RNAP^T7^‐alone, it is plausible that the lifetime of ssDNA during transcription is increased in comparison with other RNAPs (Golomb and Chamberlin, 1974; Studier and Moffatt, 1986), but mutations can be fixed in most cases. However, deletion of *ung* is likely to amplify occurrence of C deamination, thereby increasing the eventual *pyrF* inactivation rates in respect to the background (see below for analyses of different types of mutants).

When same strain set was tested for resistance to rifampicin instead of 5FOA^R^, a different pattern became apparent (Fig. 3B). First, the frequency of appearance of Rif^R^ mutants in the wild‐type hosts was lower than equivalent 5FOA^R^ clones. This was not surprising as Rif^R^ clones are expected to stem from changes in specific segments of the *rpoB* gene (encoding the essential β subunit of the housekeeping RNAP; Jatsenko *et al*., 2010), and therefore, the number of targets resulting in the resistance phenotype throughout the corresponding gene sequence ought to be lower. Second, the loss of UDG significantly increased such frequencies, in particular in the PYRC ∆*ung* strain with pSEVA221 [RNAP^T7^]. We entertain that such rise probably reflected the inherent mutagenic effect of the *ung* mutation (Duncan and Weiss, 1982; An *et al*., 2005; Algar *et al*., 2020), which could be exacerbated by the stress caused by the high expression of RNAP^T7^. Although the specific mechanisms behind the totals shown in Fig. 3A and B remain speculative, the numbers given by these experiments provided a robust baseline for appraising the effect of combining RNAP^T7^‐driven transcription with C deamination as addressed next.

### CdA‐RNAP^T7^ fusions boost diversification of DNA downstream of a T7 promoter

In order to inspect the consequences of directing CdA‐RNAP^T7^ towards the edited *pyrF*‐spanning genomic region shown in Fig. 2A and B, strains *P. putida* PYRC and *P. putida* PYRC *∆ung* expressing either rAPOBEC1‐RNAP^T7^ or pmCDA1‐RNAP^T7^ were passed through the same tests as above for quantifying emergence of both 5FOA^R^ and Rif^R^ mutants. As shown in Fig. 3C, the effect of fusions was different in each case. The wild‐type strain expressing rAPOBEC1‐RNAP^T7^ gave levels of 5FOA^R^ CFU indistinguishable from those generated by RNAP^T7^ alone. Yet, the same parameter increased ~ 60‐fold when pSEVA221 [rAPOBEC1‐RNAP^T7^] was placed in the PYRC *∆ung* host. While this result hinted that many of the mutants originated in C➔T changes, the net increase in 5FOA^R^ clones was relatively small as compared to the equivalent system devoid of CdA activity, that is expressing RNAP^T7^ only. The situation changed significantly when the rat CdA was replaced in the constructs by the lamprey deaminase pmCDA1. As shown in Fig. 3C, placing pSEVA221 [pmCDA1‐RNAP^T7^] in the PYRC strain already delivered 5FOA^R^ mutants at the same frequencies than the strain *P. putida* PYRC *∆ung* with pSEVA221 [rAPOBEC1‐RNAP^T7^]. Furthermore, when pmCDA1‐RNAP^T7^ was tested in the PYRC *∆ung* host, the frequency of mutants boosted to ~ 1.76 × 10^7^ 5FOA^R^ CFU per 10^9^ viable cells, that is ~ 1% of the whole population and > 500‐fold increase relative to the numbers corresponding to the identical strain with RNAP^T7^‐only (ca. 3.1 × 10^4^ 5FOA^R^ CFU per 10^9^ viable cells). Interestingly, as shown in Fig. 3D, the number of Rif^R^ clones detected in strains carrying the CdA variants samples changed minimally in respect to the controls (void vector and sole expression of RNAP^T7^). This suggested that introduction of the CdAs in the diversity‐generating platform of Fig. 2 increased changes in *pyrF* well above the background mutagenesis caused by the other components of the genetic device. In summary, these results indicated that [i] rAPOBEC1 and pmCDA1 fused to RNAP^T7^ enhanced diversification of target DNA sequences placed downstream of a T7 promoter, that [ii] this activity is checked by the UDG activity and that [iii] pmCDA1 was way more efficient than rAPOBEC1 as a mutagenic driver – a feature already reported when the system was tested in *E. coli* (Alvarez *et al*., 2020). One practical consequence of this is that the action of the hyperactive lamprey's variant in the wild‐type host (Fig. 3C) was equivalent to that of the rat's protein in the *∆ung* genetic background – an issue not devoid of interest for general applicability of the method (see below). The results above provided an estimation of the efficacy of the different arrangements for site‐focused mutagenesis of the *P. putida* genome. Yet, they still say little on the origin and scope of the mutations. To examine this issue, the type of changes caused by each of the CdA‐RNAP^T7^ was inspected next.

### Mutation profiles caused by CdA‐RNAP^T7^ fusions

**Fig. 4:**
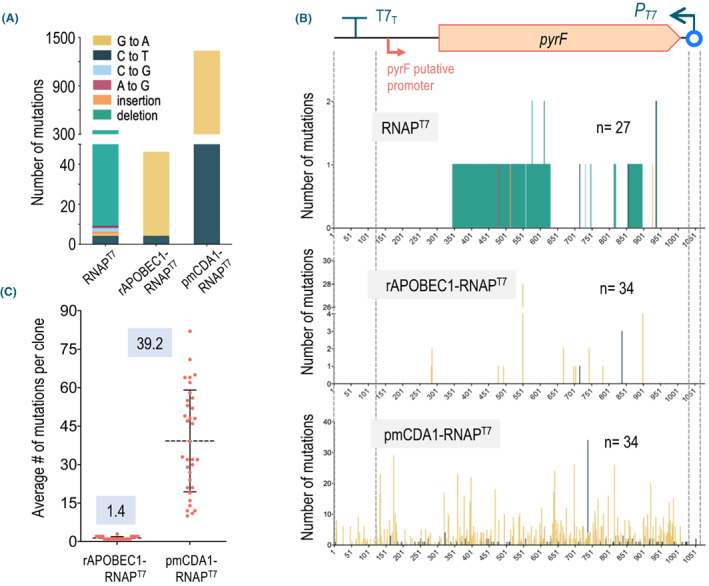
Characterization of *pyrF* mutations found in 5FOA^R^ colonies expressing CdAs. A Total number and types of mutations associated to each construct borne by *P*. *putida* PYRC *∆ung*, indicating the base substitutions found. B. Distribution and number of mutations throughout the *pyrF* segment of *P*. *putida* PYRC *∆ung* (sketched on top) in 5FOA^R^ clones carrying the construct indicated and the number of clones analysed in each case. The boundaries of the *pyrF* DNA sequence, the T7 promoter (*P*_*T7*_) and T7 terminator (T7_T_) are indicated along the location of the putative *pyrF* promoter. Note very different scales of the *Y* axis. The base changes are tagged into the coding sequence of *pyrF*. Mutations types are indicated with the same colour codes. Extension of deletions are indicated with the boundaries of the nucleotides erased from the cognate DNA. C. Average number of mutations per clone found in the 5FOA^R^ colonies analysed for each of the CdA‐RNAP^T7^ fusions. Single values are represented with red dots and means and standard deviations with black lines. Final figures are highlighted in each case. [Colour figure can be viewed at wileyonlinelibrary.com]

Figure 4 summarizes the range of mutations incorporated to the *pyrF* DNA in 5FOA^R^ clones of *P. putida* PYRC *∆ung* expressing rAPOBEC1‐RNAP^T7^ and pmCDA1‐RNAP^T7^. To generate the data shown, the genomic segment with the reporter construct engineered in the strain, that is the intervening DNA between the T7_T_ terminator and the *P*_*T7*_ promoter, was PCR‐amplified with primers out_TS1pyrF and out_TS2pyrF covering the entire DNA portion and the cognate sequences determined (see Experimental procedures). As a reference, the *pyrF* segments of 5FOA^R^ colonies of the same strain transformed with either vector‐alone pSEVA221 (12 clones) or pSEVA221 [RNAP^T7^] (27 clones) were inspected as well. Changes in the *pyrF* DNA were not detected in 5FOA^R^ mutants arising from the strain with the void vector, suggesting that the mutations mapped in another part of the genome (e.g. those determining transport or others). In contrast, we did notice a variety of changes in the same genomic segment exposed to the transcribing action of RNAP^T7^, including deletions, insertions, transitions and transversions (Fig. 4A). Despite the relatively small number of clones analysed in these controls, the results implied that transcription with RNAP^T7^ has a low‐level but still significant mutagenic effect on the DNA involved (Beletskii *et al*., 2000; Kar and Ellington, 2018; Alvarez *et al*., 2020). This is a relevant phenomenon for optimization of a general method of continuous evolution (see below).

The same type of analysis was run next on 34 5FOA^R^ clones derived from the reporter PYRC *∆ung* strains expressing each of the CdAs under examination fused to RNAP^T7^. Regardless of the CdA, all changes detected corresponded to C to T or G to A transitions occurring in both DNA strands. Interestingly, no other modifications were found, thereby enabling us to trace all changes to the action of the CdAs. The strain expressing pmCDA1‐RNAP^T7^ accumulated the highest number of transitions: a total of 1334 in all 5FOA^R^ clones, with an average of ~ 39 mutations per clone. In contrast, the strains with rAPOBEC1‐RNAP^T7^ displayed ~ 25‐fold less mutagenic activity (48 transitions detected in the clones) with just above 1 mutation per 5FOA^R^ isolate (Fig. 4B and C).

One detail of interest was that regardless of the CdA type fused to RNAP^T7^, G➔A transitions were detected more frequently than C➔T in the *pyrF* coding strand (44 vs 4 for rAPOBEC1 and 1202 vs 132 for pmCDA1 (Fig. 4A). This indicated that there is a higher mutagenic activity of the fusions on the non‐coding strand of *pyrF* in the reporter system of Fig. 2. Such a mutational bias favours changes in the non‐template strand of transcription from *P*_*T7*_ by ~ 90% vs. the template counterpart – a phenomenon observed in *E. coli* as well (Beletskii *et al*., 2000; Kar and Ellington, 2018; Moore *et al*., 2018; Alvarez *et al*., 2020). This preference might be caused by a higher exposure of the C residues of this strand to the deamination activity of the CdAs during transcription. Yet, 10% is still a notable frequency, a circumstance that plays in our favour for bringing about continuous evolution of the DNA segment of interest, as eventually every C change is in principle possible independently of the DNA strand.

Finally, in order to evaluate the efficacy of the T7 terminator (T7_T_) engineered in the reporter system for circumscribing the mutational effects of CdA‐RNAP^T7^ to the DNA segment of interest, we inspected the occurrence of C➔T and G➔A transitions beyond the nominal termination site. To this end, the *pyrF* upstream regions (i.e. beyond T7_T_) of a sample of 5FOA^R^ clones already analysed (11 from strain with pmCDA1‐RNAP ^T7^ and 17 with rAPOBEC1‐RNAP^T7^) were sequenced with primers out_TS1pyrF and pyrF_R for detection of changes indicative of CdA‐RNAP^T7^ trespassing the T7_T_ site. In the case of rAPOBEC1 fusion, no mutations beyond *pyrF* were found in the adjacent ~ 1 kb region (data not shown). On the contrary, a significant number of changes did occur in samples expressing the more active pmCDA1‐RNAP^T7^ hybrid, as shown in Fig. S4. Specifically, the *pyrF* upstream region kept an average number of mutations per clone and DNA length that was about half of those counted in the reporter gene. These figures suggested that just one T7_T_ may suffice to hinder the gross progression of the lower‐activity CdA‐RNAP^T7^ further than a cognate termination signal, but was not enough to do entirely the same with the more active counterpart (Fig. S4). This did not come as a surprise, as not less than four terminators are necessary to stop altogether transcription initiated in a T7 promoter (Moore *et al*., 2018). Regardless of the mechanisms behind differential termination activity of the T7_T_ signal with either CdA‐RNAP^T7^, these results exposed one more parameter to consider for choosing a diversity‐generating option according to specific needs (see below). But, aside of the CdA type and termination efficacy, other preferences need to be fixed as explained in the next sections.

### UDG swaps DNA strand preference for cytosine deamination

We observed a significant level of 5FOA^R^ mutants in the wild‐type *ung* ^+^ strain carrying the pmCDA1‐RNA^T7^ fusion which promoted us to inspect in more detail the type of mutations that emerged in such conditions. Since pmCDA1 has been described as a high‐activity CdA (Lada *et al*., 2011), this outcome could simply reflect that a higher C to U deamination efficiency in DNA titers out the correction mechanism mediated by the housekeeping UDG activity. But also, UDG action causes excision of U residues (Lindahl *et al*., 1977; Parikh *et al*., 1998; Schormann *et al*., 2014), leaving transient abasic sites likely to generate random replacements in the spot of the deaminated C. While this might be an unwanted occurrence when using CdAs for gene editing, it can play in our favour in applications where a large diversity of changes is pursued. With these notions in mind, we sequenced the *pyrF* DNA region of 60 5FOA^R^ clones derived from *ung* ^+^ strain *P. putida* PYRC transformed with pSEVA221 [pmCDA1‐RNAP^T7^]. Note that, as indicated above (Fig. 3), the net frequency of resistant clones was > 60‐fold lower in this UDG^+^ strain as compared to the ∆*ung* equivalent (5.68 × 10^4^ CFU vs 1.76 × 10^7^ CFU), but still considerable. Analyses of the resulting clones expectedly revealed a lower number of mutations per clone, although similar to those caused by rAPOBEC1‐RNAP^T7^ fusion in the *∆ung* strain (compare Fig. 6 vs. Fig. 4). Unexpectedly, however, the vast majority of mutations found were C➔T transitions that had taken place in the *pyrF* coding strand. Given the arrangement of the reporter construct (Fig. 2A and B), this indicated a higher deamination activity in the template strand for RNAP^T7^ transcription. This result is exactly opposite to what was found in the *∆ung* background with the same pmCDA1‐RNAP^T7^ fusion, where most changes were G➔A (Fig. 4). In addition, we found a few transversions C➔A, an occurrence that stems from filling abasic sites after cytosine deamination. Such changes were not found in any other 5FOA^R^ colony sequenced from any previous construct. Although the spatial architecture of the complex between the transcribing pmCDA1‐RNAP^T7^ and the components of the repair system is unknown, it is plausible that UDG changes the exposure of one DNA strand or the other to the deaminating activity of the fusion protein or that U residues in the template strand are less accessible to the activity of the glycosylase, but regardless of the precise process, this result adds a significant value to using pmCDA1‐RNAP^T7^ fusion in the *ung* ^+^ wild‐type strain, as a high variability can be obtained without a need to inactivate UDG. We did not fail to notice that this particular setup delivers an average of one detectable mutation per clone (Fig. 6C), what appears as a good balance between variability and function stability in the search of new phenotypes.

### Transient inhibition of native UDG boosts CdA‐RNAP^T7^ ‐mediated mutagenesis

**Fig. 5:**
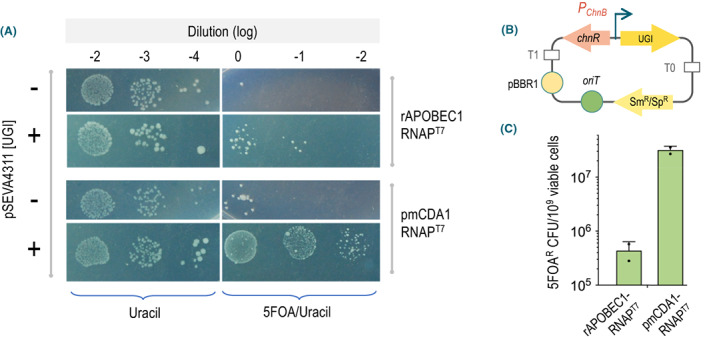
Effect of pSEVA4311[UGI] in mutagenic efficacy of CdA‐RNAP^T7^. A. Drop assay showing the frequency of 5FOA^R^ colonies of *ung* ^+^ strain *P*. *putida* PYRC with plasmids expressing separately rAPOBEC1‐RNAP^T7^ or pmCDA1‐RNAP^T7^ and added or not with compatible plasmid pSEVA4311[UGI] as indicated in each case. B. Schematic representation of the functional parts of pSEVA4311 [UGI], not to scale. This is a medium‐copy number plasmid with the origin of replication of pBBR1, an *oriT* for conjugative mobilization, an Sm^R^/Sp^R^ gene for selection and a ChnR/*P*_*ChnB*_ expression system driving transcription of the UGI gene. C. Frequency of 5FOA^R^ clones generated from *P*. *putida* expressing the CdA‐RNAP^T7^ variants indicated and added with of pSEVA4311[UGI]. Compare these figures with those of the same strains without UGI shown in Fig. 3C. Adding or not the ChnR inducer cyclohexanone to the cultures had no significant effect on the reported frequencies (not shown). The mean (bars), standard deviation (lines) and single values (dots) of two independent biological replicas are shown. [Colour figure can be viewed at wileyonlinelibrary.com]

As shown above, the lack of UDG is key for enabling C deamination to turn into conversion to a T residue in DNA. In pragmatic terms, this means that the hosts for optimal operation of CdA‐RNAP^T7^ fusions should lack the cognate *ung* gene. In most cases, deletion of the corresponding DNA can be easily entered in the genome of the strain at issue, but in other instances, the loss of UDG can interfere with the outcome of the pursued diversification. In these instances, it is possible to inhibit the enzyme *in vivo* through co‐expression of an uracil‐DNA glycosylase inhibitor (UGI) protein. The most used UGI comes from the bacteriophage PBS2 (Katz *et al*., 1976; Cone *et al*., 1980; Wang and Mosbaugh, 1989), and it consists of a thermostable and highly acidic small peptide (84 amino acids, 9.4 kDa), which binds reversibly to UDG in 1:1 molar stoichiometry (Wang and Mosbaugh, 1989). To leverage this UGI for our purposes, the cognate DNA sequence was optimized for expression in *Pseudomonas* (Gallagher *et al*., 2021) and cloned in medium‐copy number Sm^R^/Sp^R^ vector pSEVA4311 (see details in Experimental Procedures). The resulting UGI^+^ plasmid was then passed through conjugation to *ung* ^+^ strain *P. putida* PYRC already bearing either pSEVA221 [rAPOBEC1‐RNAP^T7^] or pSEVA221 [pmCDA1‐RNAP^T7^], and the exconjugant strains were assayed for emergence of 5FOA^R^ colonies as before. The results (Fig. 5) showed that incorporation of pSEVA4311 [UGI] to PYRC strain increased mutagenic efficiency of rAPOBEC1‐RNAP^T7^ on *pyrF* by ~ 250‐fold (ca. 4.3 × 10^5^ CFU) and that of pmCDA1‐RNAP^T7^ by ~ 1000‐fold (ca. 3.1 × 10^7^ CFU) in respect to the conditions lacking UGI (1.9 × 10^3^ CFU and 5.7 × 10^4^ CFU, respectively). Inspection of the data of Fig. 5 in fact suggests that ectopic expression of UGI brought about the same mutant frequencies as those observed in PYRC ∆*ung* strains (i.e. with an *ung* deletion). It is thus feasible to induce a transient UDG‐minus phenotype by addition of compatible construct pSEVA4311 [UGI] to the cells already loaded with plasmids expressing CdA‐RNAP^T7^ variants for the sake of boosting diversification of the target gene. To test whether the mutation profile of each DNA strand involved was the same as in the PYRC *∆ung* strain, a number of 5FOA^R^ clones of the *P. putida* PYRC reporter strain expressing both pmCDA1‐RNAP^T7^ and the UGI peptide were sequenced. The results shown in Fig. S5 indicated that – similarly to the situation in a genetic context lacking UDG –deamination occurred preferentially in the non‐template strand. This occurrence widens the range of conditions and strains that can be chosen for particular DNA diversification assignments.

**Fig. 6:**
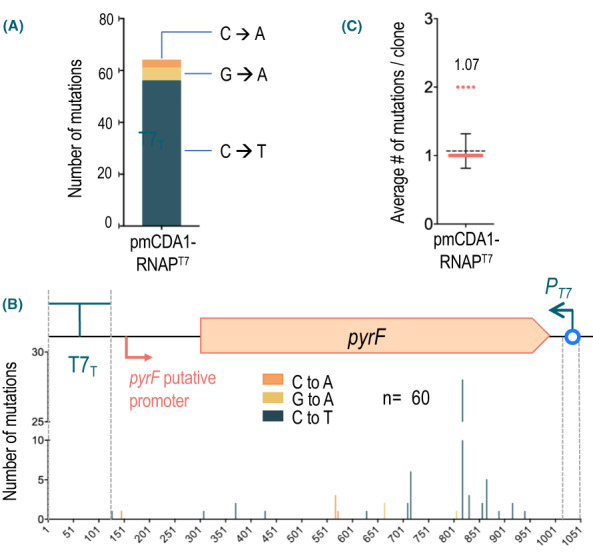
Characterization of *pyrF* mutations found in 5FOA^R^ colonies of UDG^+^ strain *P*. *putida* PYRC expressing the pmCDA1‐RNAP^T7^ fusion. A. Total number of mutations identified in the 60 clones analysed, with an indication of the base substitutions found. B. Average number of mutations identified per sequenced clone. Single values are represented with red dots and means and standard deviations with black lines. C. Type and position of each mutation in the *pyrF* gene identified in the 60 5FOA^R^ clones sequenced. The boundaries of the T7 promoter (*P*_*T7*_), T7 terminator (T7_T_) and the putative *pyrF* promoter are indicated. The base changes shown correspond to the coding sequence of *pyrF*. The different types of mutations labelled with the same colour codes as in (A). [Colour figure can be viewed at wileyonlinelibrary.com]

## Conclusion

The work presented in this article provides the genetic tools and the variables to consider for setting continuous diversification of any DNA segment of the genome of *P. putida*. Since all constructs are borne by broad‐host range plasmids, it is plausible that the very same platform can be effectively reused in other Gram‐negative bacteria. Specifically, by using a *pyrF*‐based reporter system, the efficacy of each CdA‐RNAP^T7^ fusion as mutagenic agent has been determined, the role of UDG in the process settled and the efficacy of a T7 terminator to restrain DNA diversification beyond a prefixed site documented. Given the degeneracy of the genetic code and that not every base change necessarily originates an inactive *pyrF* mutant, it is likely that the actual DNA diversification figures are at least threefold higher than the ones shown throughout this work. As indicated above, the desired mutagenesis levels can be modulated at will by picking one of the fusions available and playing with having either *ung* ^*−*^ or *ung* ^+^ strains as hosts of the process – or engineering a transient UDG‐minus phenotype upon addition of a compatible plasmid expressing the inhibitory peptide UGI. Although not tested directly in this work, the length of the genomic segment to be diversified could in principle be limited by engineering a series of terminators or a dCas9‐based device (Alvarez *et al*., 2020) to inhibit the advancing CdA‐RNAP^T7^. In other cases, such *rowing base editors* can be let to proceed through longer DNA segments in the genome if desired. In sum, we believe that the hereby described devices may become a phenomenal addition to the already rich toolset available for many types of bioengineering endeavours in *P. putida* (Martínez‐García and de Lorenzo, 2017; Martin‐Pascual *et al*., 2021).

## Experimental procedures

### Strains, plasmids, growth conditions and general techniques

The list of bacterial strains and plasmids used in this study can be found in Tables S1, S2 and S3. The *P. putida* strains employed in this work were derived from variant EM42 (Martinez‐Garcia *et al*., 2014) a genome‐edited derivative of reference wild‐type isolate KT2440 (Nelson *et al*., 2002). *E. coli* strains DH5α, CC118 (Hanahan and Meselson, 1983; Manoil and Beckwith, 1985) and their λ*pir* derivatives were used as hosts of intermediate constructs. The presence of λ*pir* allows proliferation of plasmids with an R6K *oriV* origin of replication (e.g. pEMG). Bacteria were regularly grown in liquid or solid (1.5% agar) LB medium at 37°C for *E*. *coli* and 30°C for *P. putida*. Where indicated, plates of M9 minimal medium (Sambrook *et al*., 1989) were supplemented with 0.2% (w/v) sodium citrate as the sole carbon source and added with 5‐fluoroorotic acid (5FOA, 250 μg ml^−1^) and/or uracil (ura, 20 μg ml^−1^) as required. Antibiotics were added to liquid or solid media at various concentrations: streptomycin (Sm, 50 μg ml^−1^ for *E. coli* and 100 μg ml^−1^ for *P. putida*), kanamycin (Km, 50 μg ml^−1^) and rifampicin (Rif, 100 μg ml^−1^). DNA manipulations followed standard laboratory techniques (Sambrook *et al*., 1989). Plasmids were constructed with either classical cloning procedures or Gibson assembly (Gibson *et al*., 2009; Gibson *et al*., 2010). PCR reactions were run with Q5 High Fidelity polymerase (New England Biolabs, Ipswich, MA, USA) according to manufacturer's instructions. Diagnostic PCRs were performed using Green MasterMix (BioTools, Madrid, Spain) taking fresh single colonies as the starting material. *P. putida* cells were transformed as described by Choi and Schweizer (2006)) in a Gene Pulser/Pulse Controller (Bio‐Rad, Hercules, California, USA) system with 2.5 kV, 25 μF, 200 Ω. Tri‐parental matings (Kessler *et al*., 1992) for mobilization of plasmids from *E. coli* strains to *P*. *putida* were performed with a simplified protocol (Sambrook *et al*., 1989). For CFU counting, culture dilutions were plated on either LB or M9 minimal media with 0.2% citrate and adequate supplements for isolating individual clones as indicated in each case.

### Construction of deletion and insertion mutants in *P. putida* strains

The method for inserting the reporter PYRC segment into the genome of *P. putida* was adapted from Martinez‐Garcia and de Lorenzo (2011); Fig. S1). In brief, a ~ 1 kb DNA fragment flanked by homology regions surrounding the native *pyrF* locus was synthesized (Genecust™, Ellange, Luxemburg) and added to pEMG as an EcoRI and BamHI insert, thereby originating pEMG [PYRC]. This plasmid was then transformed to strain *P. putida* EM42 *∆pyrF* and consequently plated on LB agar plates supplemented with Km to isolate co‐integration events. Then, pSW‐I plasmid controlling expression of I‐SceI endonuclease was also electroporated into selected clones. This enzyme, induced by addition of 3‐methyl benzoate, triggered the second recombination event by cleaving the chromosome at its specific targets included in the pEMG sequence. Finally, Km‐sensitive clones were isolated and checked with colony PCR with primers out_TS1pyrF (GCCGCTGTTCGCCAGTCTGTCG) and out_TS2pyrF (GATCGACTACAAGGCCGAAGACGTGC) to verify the correct modification in the genome vs. reversion to the wild‐type genotype. pSW‐I plasmid was then cured by several growth passages with no antibiotic pressure. The ensuing strain was called *P. putida* PYRC. Deletion of the *ung* gene of *P. putida* was done as explained in (Martinez‐Garcia and de Lorenzo, 2011) and verified with diagnostic PCR oligos ung‐junction‐F (CATCGCGGGCATTGATCG) and ung‐junction‐R (GAAGTGCGTC AGCGGTCC).One correct clone was also cured of pSW‐I plasmid and named *P. putida* PYRC *∆ung*. All plasmids used in these procedures are listed in Table S3.

### Expression of cytosine deaminases fused to RNAP^T7^ fusions and mutagenesis tests

Plasmids encoding the two CdA‐RNAP^T7^ used in this work have been described elsewhere (Alvarez *et al*., 2020). In them, either rat apolipoprotein B mRNA editing enzyme 1 (rAPOBEC‐1) or *Petromyzon marinus* (lamprey) cytidine deaminase 1 (PmCDA‐1) is respectively fused to the N‐terminus domain of the RNAP^T7^ by a (G_3_S)_7_ linker (28 aa in total). In addition, they both bear a T7 tag (an epitope composed of an 11‐residue peptide from the leader sequence of the T7 phage gene 10) added to the N‐terminus of each of the CdA sequences and the fusions are transcribed through a TetR/P_tetA_ expression device (Lederer *et al*., 1996). Control plasmids, empty pSEVA221 and pSEVA221 [RNAP^*T7*^] are described in Table S3. Plasmids were separately entered into reporter *P*. *putida*, and strains with the different constructs grown overnight at 30°C in liquid LB with Km. Cultures were then diluted in the same medium to an OD_600_ of 0.02 and regrown with shaking to an OD_600_ of 0.4 to 0.7. Cells were then washed with PBS 1x and serial dilutions of 100 μL plated on M9 medium with Ura (for viability) and with Ura + 5FOA for selecting 5FOA^R^ mutants. After overnight growth, CFU were counted from each plate and the mutation rate was calculated dividing the count of CFU in M9/Ura/5FOA plates by CFU in M9/Ura plates and normalizing figures to 10^9^ viable cells. The off‐target activity of the system was calculated by plating the same cells on LB/Rif media, and Rif^R^ clones were also normalized to 10^9^ viable cells.

### Sequencing and analysis of cytosine deaminases‐induced mutations

5FOA^R^ colonies were used as starting material for colony PCR reactions with primers out_TS1pyrF and out_TS2pyrF (see above) which amplified a ~ 2.2 kb fragment covering the reporter *pyrF* region engineered in *P*. *putida* PYRC (Fig. 2) and ~ 500 bp upstream, thereby encompassing also ORFs PP1813 and PP1814. Amplified DNAs were directly purified with ExoSap‐IT™ PCR Product Cleanup Reagent (Thermo Fisher Scientific, Applied Biosystems) and submitted to Sanger sequencing with primers pT7p (TAATACGACTCACTATAGGG) and pT7t (ACCCC TCAAGACCCGTTTAG) to address the type of mutations in the *pyrF* locus or with primers out_TS1pyrF (GCCGCTGTTCGCCAGTCTGTCG) and pyrF_R (TCACGCCAATCAGCAACG) to analyse the mutations upstream of T7_T_. Sequences were analysed manually with SeqMan Pro™ DNASTAR software and compared against the sequence of the modified *pyrF* segment as reference. Results were plotted using GraphPad Prism 6 (GraphPad Software Inc., San Diego, CA, USA).

### Construction and utilization of UGI expression plasmid

In order to build a construct for entering UGI activity in *P*. *putida*, the ChnR‐*P*_*Chnb*_ promoter was excised from pSEVA2311 as a PacI/AvrI fragment and inserted into the same sites of pSEVA427 resulting in pSEVA4211‐gfp (Table S3). The DNA sequence encoding UGI was PCR‐amplified from pPSV39‐UGI with oligonucleotides p4211‐UGI‐F (CGCGAATTCCACGGGAGGAAAGATGACG) and p4211‐UGI‐R (CTATCAACAGGAGTCCAA GACTAGTTTAGAGCATCTTGTTTTGTTCTC) and cloned through Gibson assembly into EcoRI/SpeI‐digested pSEVA4211‐gfp, yielding pSEVA4211[UGI]. Then, the pBBR1 *oriV* from pSEVA131 was isolated as a FseI‐AscI fragment and cloned into similarly digested pSEVA4211 [UGI] to generate the Sm^R^ plasmid pSEVA4311 [UGI], which is compatible with pSEVA221 [rAPOBEC1‐RNAP^T7^] and pSEVA221 [pmCDA1‐RNAP^T7^]. pSEVA4311 [UGI] was then passed to the *P*. *putida* strains indicated in each case through tri‐parental mating as explained above. In order to test the effect of UGI expression on mutagenic efficacy of the CdA‐RNAP^T7^ fusions, cultures of the thereby constructed strains were grown overnight at 30°C in LB supplemented with Km and Sm to secure retention of both plasmids. Each of the mutagenic fusions cells were regrown in fresh media, let grow to mid‐exponential phase and processed and plated on selective media to assess viability and emergence 5FOA^R^ CFU as explained before.

## Conflict of interest

The authors declare no conflict of interest.

## Supporting information


**Table S1.** *E. coli* strains used in this work.
**Table S2.** *Pseudomonas putida strains* used in this work.
**Table S3.** Plasmids used and constructed in this work.
**Fig. S1.** Refactoring the *pyrF* region of *P. putida* as a reporter of mutagenic activity of CdA‐RNAP ^T7^ fusions. (A) Organization of the genomic region of interest in the starting strain *P. putida* EM42 *ΔpyrF* 2018. (B) Arrangement of delivery plasmid pEMG [PYRC] (not to scale). Vector pEMG (Martinez‐Garcia and de Lorenzo, 2011) was inserted with a synthetic DNA fragment composed by the *P. putida pyrF* gene bordered by a T7 terminator (T_7T_) at 5’ and a T7 promoter (*P_T7_*) at 3’ along with flanking 500 pb of DNA homologous to either side of the native genomic sequence. The cassette was then introduced in *P. putida* EM42 *ΔpyrF* by recombination following the protocol described in Experimental Procedures. (C) After resolution of the cointegrate, the resulting strain was named *P. putida* PYRC, which carried the PYRC reporter segment in its genome as indicated at the bottom.
**Fig. S2.** Characterization of *P. putida* reporter strains. Viability of reporter and parental strains in minimal medium M9/Citrate, M9/Citrate supplemented with uracil and M9/Citrate supplemented with uracil and 5FOA. Each culture was grown overnight and then normalized to OD600 = 1 in PBS 1x. Series of ten‐fold dilutions of each culture were prepared and 5 μl drops of each dilution were plated.
**Fig. S3.** Performance of the *tetR/P*_*tetA*_ expression device in *P. putida* EM42. (A) Fluorescent cell cytometry of *E. coli* CC118 and *P. putida* PYRC bearing plasmid pS221 Ptet‐GFP^1^. The pictures show^2^ the distribution of fluorescence in populations under non‐inducing conditions (*t* = 0 h and No aTc) and after induction at the indicated time points with 0.5 μM aTc (*t* = 1 h and 2 h). The region considered negative for the fluorescence signal is marked with a grey dashed line, as assessed by control cells carrying an empty pSEVA221 plasmid (purple plot). At least 80.000 events were analyzed in each sample. (B) Visual inspection of GFP fluorescence signal in the same samples under blue light. Note a regulated, aTc‐inducible expression of GFP in *E. coli* in contrast with a virtually constitutive expression in *P. putida*. Anhydrotetracycline (aTc); arbitrary units (a.u.)
**Fig. S4.** Mutagenic activity of advancing CdA‐RNAP ^T7^ fusions beyond a T7 termination signal. The figure summarizes the characterization of mutations upstream of the *pyrF* gene of *P. putida* PYRC *Δung* borne by 5FOA^R^ colonies expressing the pmCDA1‐RNAP^T7^ fusion. (A) Number and frequency of mutations found through upstream region of the PYRC cassette of 11 5FOA^R^ colonies. Adjacent PP1813 and PP1814 genes are shown. Different types of mutations are indicated with a color code. (B) Frequency of transitions per clone and nucleotide caused by pmCDA1‐RNAP^T7^ on the PYRC segment proper (on target) and the upstream region beyond the terminator. (C) Average number of mutations per clone found in the upstream region of the PYRC segment of the 5FOA^R^ colonies. Single values are represented with red dots and means and standard deviations with black lines. (D) Total number of mutations and base substitutions found in the upstream region shown.
**Fig. S5.** Non‐template strand preference is recovered in UGI expressing clones. (A) Total number and types of mutations borne by *P. putida* PYRC expressing pmCDA1‐RNAP^T7^ and UGI. (B) Average number of mutations per clone found in the 5FOA^R^ colonies analyzed. Single values are represented with red dots and means and standard deviations with black lines. (C) Distribution and number of mutations throughout the *pyrF* segment of *P. putida* PYRC in 28 5FOA^R^ clones carrying the construct indicated previously. The boundaries of the *pyrF* DNA sequence, the T7 promoter (*P_T7_*) and T7 terminator (T_7T_) are indicated along the location of the putative *pyrF* promoter. The base changes are tagged into the coding sequence of *pyrF*. Mutations types are indicated with the same color codes.
