# Supplementary material for: Hypermutation of specific genomic loci of Pseudomonas putida for continuous evolution of target genes
Source: Microb Biotechnol. 2022 Jun 13;15(9):2309–23. doi: 10.1111/1751-7915.14098 (PMC9437889; doi:10.1111/1751-7915.14098)
Supplement: Supplementary file 1 — Table S1. E. coli strains used in this work. Table S2. Pseudomonas putida strains used in this work. Table S3. Plasmids used and constructed in this work. Fig. S1. Refactoring the pyrF region of P. putida as a reporter of mutagenic activity of CdA‐RNAP T7 fusions. (A) Organization of the genomic region of interest in the starting strain P. putida EM42 ΔpyrF 2018. (B) Arrangement of delivery plasmid pEMG [PYRC] (not to scale). Vector pEMG (Martinez‐Garcia and de Lorenzo, 2011) was inserted with a synthetic DNA fragment composed by the P. putida pyrF gene bordered by a T7 terminator (T7T) at 5’ and a T7 promoter (PT7 ) at 3’ along with flanking 500 pb of DNA homologous to either side of the native genomic sequence. The cassette was then introduced in P. putida EM42 ΔpyrF by recombination following the protocol described in Experimental Procedures. (C) After resolution of the cointegrate, the resulting strain was named P. putida PYRC, which carried the PYRC reporter segment in its genome as indicated at the bottom. Fig. S2. Characterization of P. putida reporter strains. Viability of reporter and parental strains in minimal medium M9/Citrate, M9/Citrate supplemented with uracil and M9/Citrate supplemented with uracil and 5FOA. Each culture was grown overnight and then normalized to OD600 = 1 in PBS 1x. Series of ten‐fold dilutions of each culture were prepared and 5 μl drops of each dilution were plated. Fig. S3. Performance of the tetR/P tetA expression device in P. putida EM42. (A) Fluorescent cell cytometry of E. coli CC118 and P. putida PYRC bearing plasmid pS221 Ptet‐GFP1. The pictures show2 the distribution of fluorescence in populations under non‐inducing conditions (t = 0 h and No aTc) and after induction at the indicated time points with 0.5 μM aTc (t = 1 h and 2 h). The region considered negative for the fluorescence signal is marked with a grey dashed line, as assessed by control cells carrying an empty pSEVA221 plasmid (purple plot). At least 80.000 events [file MBT2-15-2309-s001.pdf]

Supplementary Information to Velazquez *et al.*

Supplementary Table S1. *E. coli* strains used in this work.

Strains	Use	Genotype	Reference
CC118	Cloning host	*F- Δ(ara-leu), araD, ΔlacX74, galE, galK, phoA, thi1, rpsE, rpoB, argE (Am), recA1*	(Manoil and Beckwith, 1985)
DH5α	Cloning host	*F-, supE44, ΔlacU169, (φ80 lacZDM15), hsdR17, (rkmk+), recA1, endA1, thi1, gyrA, relA*	(Hanahan and Meselson, 1983)
DH5α λ*pir*	Cloning host for suicide plasmids (R6K origin of replication)	λ*pir* phage lysogen of DH5α	Lab Collection
HB101	Helper strain for tripartite conjugation	*F⁻ λ⁻ hsdS20(rB⁻ mB⁻) recA13 leuB6(Am) araC14 Δ(gpt-proA)62 lacY1 galK2(Oc) xyl-5 mtl-1 thiE1 rpsL20 glnX44B(AS)B*	(Boyer and Roulland-Dussoix, 1969)

Supplementary Table S2. *P. putida* strains used in this work.

Strains	Description	Reference
EM42	Cell factory strain derived from KT2440. Δprophage1, Δprophage4, Δprophage3, Δprophage2, ΔTn7, ΔendA-1, ΔendA-2 ΔhsdRMS, Δflagellum, ΔTn4652	(Martinez-Garcia et al., 2014)
EM42 Δ*pyrF*	EM42 derivative with full deletion of *pyrF* gene	(Aparicio et al., 2018)
PYRC	EM42 Δ*pyrf* derivative strain with reporter *pyrF* cassette engineered in the *pyrF* locus	This work
PYRC Δ*ung*	PYRC derivative with full deletion of *ung* gene	This work

Supplementary Table S3. Plasmids used and constructed in this work

Plasmid	Description	Reference
pRK600	Helper plasmid for conjugation; *oriV* (ColE1), RK2 (*mob*+ *tra*+); pRK2013 derivative (Ditta et al., 1980); Cm^R	(Kessler et al., 1992)
pSEVA131	Standard SEVA expression vector; *oriV* (pBBR1); Ap^R	(Silva-Rocha et al., 2013)
pSEVA427	Standard SEVA expression vector; *oriV* (pBBR1); gfp gene as cargo; Sm^R/Sp^R	(Silva-Rocha et al., 2013)

pEMG	Plasmid used for deletions; *lacZα* with two flanking I-SceI target sites; *oriV* (R6K); Km^R	(Martinez-Garcia and de Lorenzo, 2011)
pSW-I	Expression plasmid for I-SceI restriction enzyme under XylS-P_m promoter control; *oriV* (RK2); Ap^R	(Wong and Mekalanos, 2000)
pUC57 [PYRC]	pUC57 plasmid bearing a synthetic DNA sequence consisting of the *P. putida pyrF* gene with its putative promoter flanked by a T7 terminator ($T7_T$) at 5' and a T7 promoter (P_{T7}) at 3'. It also incorporates 500 bp-homologous regions at the sides of the native *pyrF* gene of *P. putida* for enabling recombination of the $T7_T$-*pyrF*-P_{T7} construct into the native *pyrF* locus.	GeneCust (Ellange, Luxemburg)
pEMG [PYRC]	pEMG derivative bearing the PYRC segment as an EcoRI/BamHI insert	This work
pEMG-*ung*	pEMG derivative bearing an EcoRI/BamHI insert with homologous regions to the flanking sequences of the *P. putida ung* gene	(Algar et al., 2020)
pSEVA221	Standard SEVA expression vector; *oriV* (RK2); Km^R	(Silva-Rocha et al., 2013)
pSEVA221 [$RNAP^{T7}$]	pSEVA221 derivative with $RNAP^{T7}$ gene under *tetR*-P_{tet} expression device; *oriV* (RK2); Km^R	(Alvarez et al., 2020)
pSEVA221 [rAPOBEC1-$RNAP^{T7}$]	pSEVA221 derivative with rat APOBEC-1 fused to N-terminus of $RNAP^{T7}$ under *tetR*-P_{tet} expression device; *oriV* (RK2); Km^R	(Alvarez et al., 2020)
pSEVA221 [pmCDA1-$RNAP^{T7}$]	pSEVA221 derivative with lamprey pmCDA-1 fused to N-terminus of $RNAP^{T7}$ under *tetR*-P_{tet} expression device; *oriV* (RK2); Km^R	(Alvarez et al., 2020)
pSEVA221 [AID-$RNAP^{T7}$]	pSEVA221 derivative with human AID fused to N-terminus of $RNAP^{T7}$ under *tetR*-P_{tet} expression device; *oriV* (RK2); Km^R	(Alvarez et al., 2020)
pS221-ptetGFP	pSEVA221 derivative with a *msf*GFP gene under *tetR*-P_{tet} expression device; *oriV* (RK2); Km^R	This work
pSEVA4211-gfp	pSEVA427 derivative with the chnR-P_{ChnB} expression device cloned as PacI/AvrII insert	This work
pPSV39-UGI	pPSV39 derivative with UGI ORF optimized for expression in *Pseudomonas* cloned as a SacI/XbaI fragment	Joseph Mougous Lab (Gallagher et al., 2021)
pSEVA4211 [UGI]	pSEVA4211-gfp derivative with UGI cloned into EcoRI/SpeI target sites.	This work
pSEVA4311 [UGI]	pSEVA4211 [UGI] derivative with a pBBR1 *oriV*.	This work

Supplementary Fig. S1. Refactoring the *pyrF* region of *P. putida* as a reporter of mutagenic activity of CdA-RNAP^{T7} fusions.

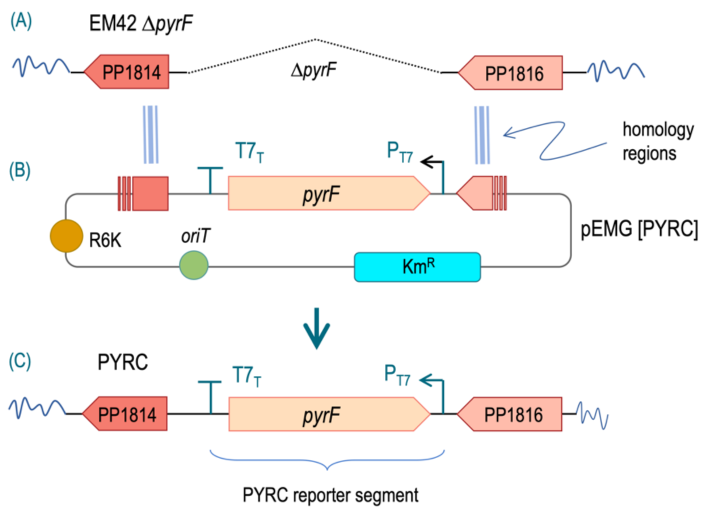

(A) Organization of the genomic region of interest in the starting strain *P. putida* EM42 Δ*pyrF* [60]. (B) Arrangement of delivery plasmid pEMG [PYRC] (not to scale). Vector pEMG (Martinez-Garcia and de Lorenzo, 2011) was inserted with a synthetic DNA fragment composed by the *P. putida pyrF* gene bordered by a T7 terminator ($T7_T$) at 5' and a T7 promoter (P_{T7}) at 3' along with flanking 500 pb of DNA homologous to either side of the native genomic sequence. The cassette was then introduced in *P. putida* EM42 Δ*pyrF* by recombination following the protocol described in Experimental Procedures. (C) After resolution of the cointegrate, the resulting strain was named *P. putida* PYRC, which carried the PYRC reporter segment in its genome as indicated at the bottom.

Supplementary Fig. S2. Characterization of *P. putida* reporter strains.

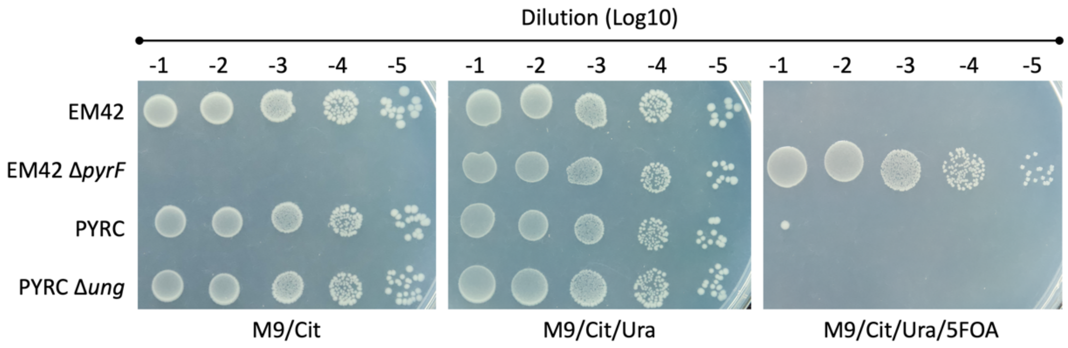

Viability of reporter and parental strains in minimal medium M9/Citrate, M9/Citrate supplemented with uracil and M9/Citrate supplemented with uracil and 5FOA. Each culture was grown overnight and then normalized to OD_{600} = 1 in PBS 1x. Series of ten-fold dilutions of each culture were prepared and 5 μl drops of each dilution were plated.

Supplementary Fig. S3. Performance of the *tetR/P*$_{tetA}$ expression device in *P. putida* EM42

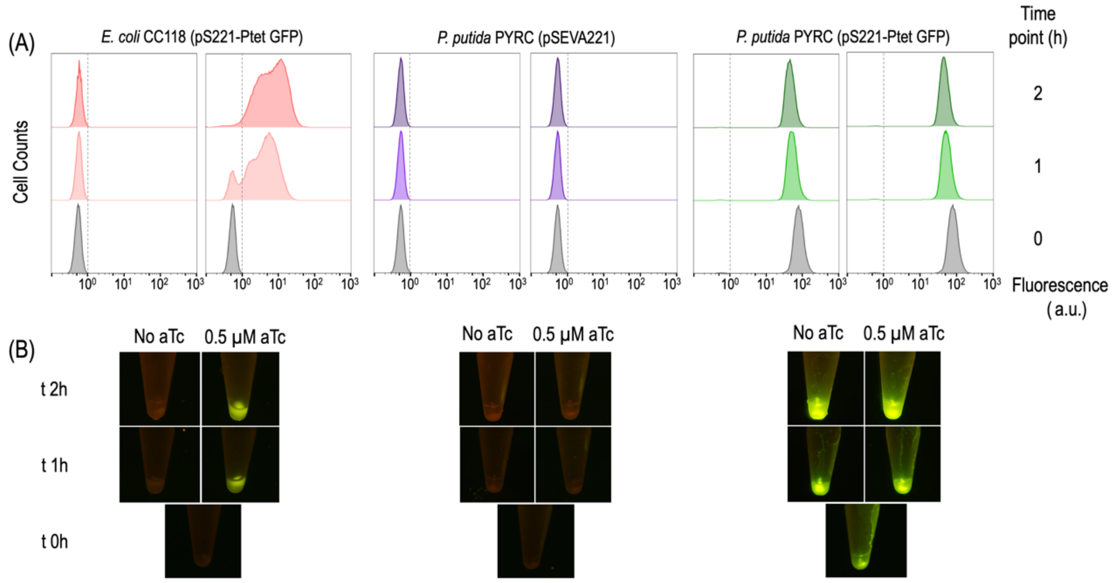

(A) Fluorescent cell cytometry of *E. coli* CC118 and *P. putida* PYRC bearing plasmid pS221 Ptet-GFP[1]. The pictures show[2] the distribution of fluorescence in populations under non-inducing conditions (t = 0 h and No aTc) and after induction at the indicated time points with 0.5 µM aTc (t = 1 h and 2 h). The region considered negative for the fluorescence signal is marked with a grey dashed line, as assessed by control cells carrying an empty pSEVA221 plasmid (purple plot). At least 80.000 events were analyzed in each sample. (B) Visual inspection of GFP fluorescence signal in the same samples under blue light. Note a regulated, aTc-inducible expression of GFP in *E. coli* in contrast with a virtually constitutive expression in *P. putida*. Anhydrotetracycline (aTc); arbitrary units (a.u.)

1 For construction of this plasmid, a 760 bp segment from pSEVA238M was excised as a XbaI/SpeI-fragment and cloned in the same sites of digested pSEVA221[AID-RNAP^{T7}] (Supplementary Table S3).

2 Either *E. coli* CC118 or *P. putida* PYRC were transformed with either pS221 Ptet-GFP or pSEVA221 as indicated, grown overnight in filtered LB medium with Km (30°C in the case of *P. putida* and 37°C in the case of *E. coli*), diluted to an OD_{600} of 0.05 and regrown until an OD_{600} of ~ 0.3. Then, each culture was split and samples induced or not with aTc. This time point was considered t= 0 h and one aliquot of each sample harvested and analyzed to set the basal level of expression. 1 mL aliquots were later retrieved at two different time points spun down in a tabletop centrifuge (13,000 rpm for 1 min), washed twice with filtered PBS 1x and diluted to an OD_{600} < 0.1. Cells were stored on ice and immediately analyzed with a MACS-QuantTM VYB cytometer (Miltenyi Biotec GmbH). GFP was excited at 488 nm, and the fluorescence signal was recovered with a 525/40 nm band pass filter. Data processing was carried out using the FlowJoTM software (www.flowjo.com). GFP quantification was tested by firstly gating cells in a side scatter against forward scatter plot. Each experiment was repeated twice.

Supplementary Fig. S4. Mutagenic activity of the advancing pmCDA1-RNAP^{T7} fusion beyond a T7 termination signal.

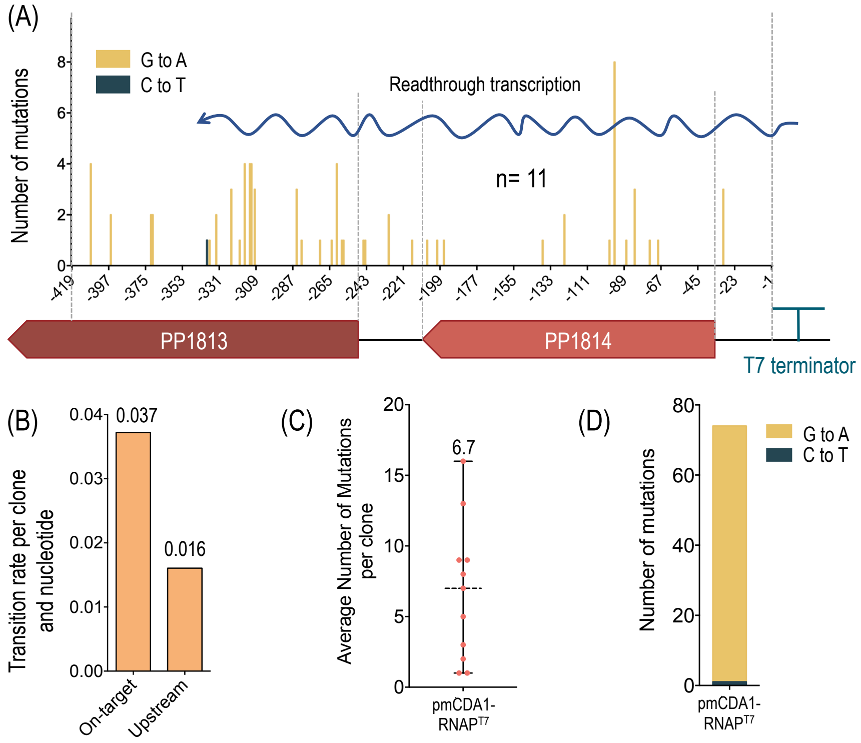

The figure summarizes the characterization of mutations upstream of the *pyrF* gene of *P. putida* PYRC Δ*ung* borne by 5FOA^R colonies expressing the pmCDA1-RNAP^{T7} fusion. (A) Number and frequency of mutations found through upstream region of the PYRC cassette of 11 FOA^R colonies. Adjacent PP1813 and PP1814 genes are shown. Different types of mutations are indicated with a color code. (B) Frequency of transitions per clone and nucleotide caused by pmCDA1-RNAP^{T7} on the PYRC segment proper (on target) and the upstream region beyond the terminator. (C) Average number of mutations per clone found in the upstream region of the PYRC segment of the 5FOA^R colonies. Single values are represented with red dots and means and standard deviations with black lines. (D) Total number of mutations and base substitutions found in the upstream region shown.

Supplementary Fig. S5. Non-template strand preference is recovered in UGI expressing clones

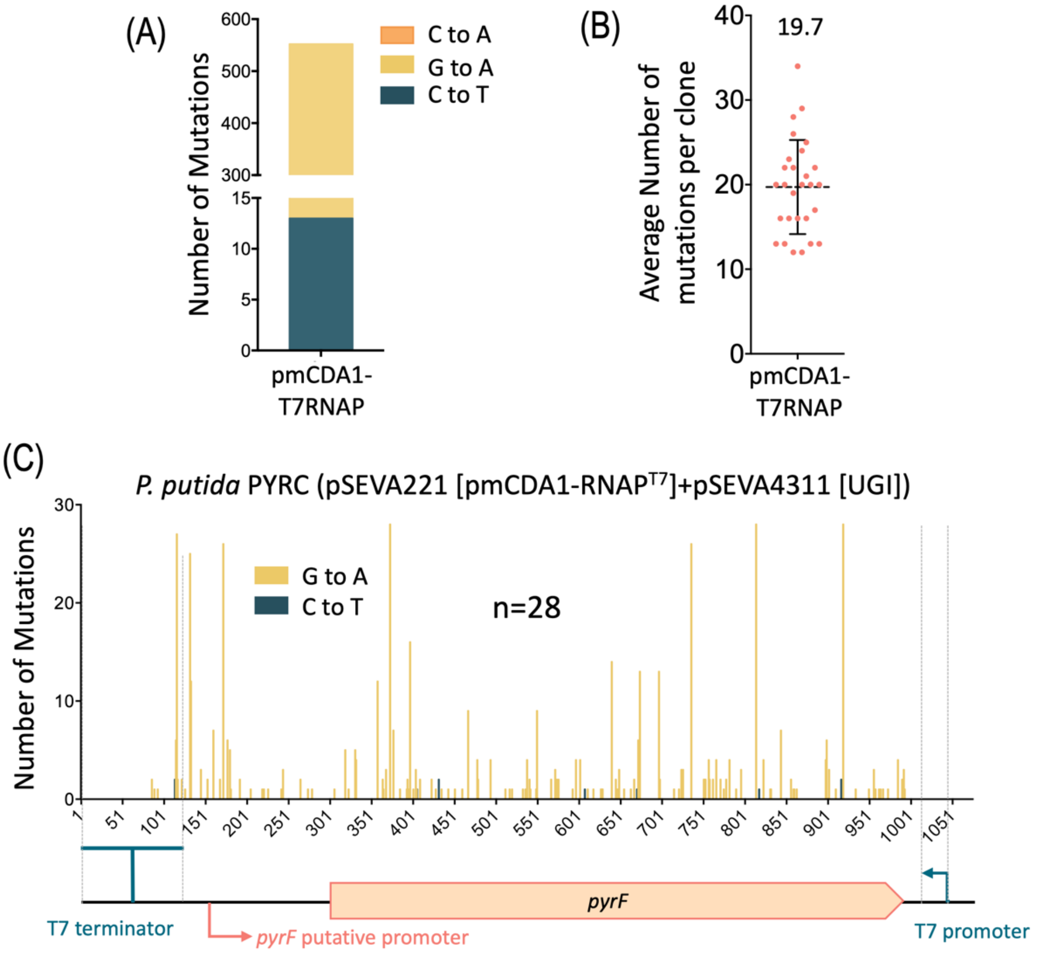

(A) Total number and types of mutations borne by *P. putida* PYRC expressing pmCDA1-RNAP^{T7} and UGI. (B) Average number of mutations per clone found in the 5FOA^{R} colonies analyzed. Single values are represented with red dots and means and standard deviations with black lines. (C) Distribution and number of mutations throughput the *pyrF* segment of *P. putida* PYRC in 28 5FOA^{R} clones carrying the construct indicated previously. The boundaries of the *pyrF* DNA sequence, the T7 promoter (P_{T7}) and T7 terminator ($T7_{T}$) are indicated along the location of the putative *pyrF* promoter. The base changes are tagged into the coding sequence of *pyrF.* Mutations types are indicated with the same color codes.

REFERENCES

Algar, E., Al-Ramahi, Y., de Lorenzo, V., and Martinez-Garcia, E. (2020) Environmental performance of *Pseudomonas putida* with a uracylated genome. *Chembiochem* **21**: 3255-3265.

Alvarez, B., Mencia, M., de Lorenzo, V., and Fernandez, L.A. (2020) In vivo diversification of target genomic sites using processive base deaminase fusions blocked by dCas9. *Nat Commun* **11**: 6436.

Aparicio, T., de Lorenzo, V., and Martinez-Garcia, E. (2018) CRISPR/Cas9-Based counterselection boosts recombineering efficiency in *Pseudomonas putida. Biotechnol J* **13**: e1700161.

Boyer, H.W., and Roulland-Dussoix, D. (1969) A complementation analysis of the restriction and modification of DNA in *Escherichia coli. J Mol Biol* **41**: 459-472.

Ditta, G., Stanfield, S., Corbin, D., and Helinski, D.R. (1980) Broad host range DNA cloning system for gram-negative bacteria: construction of a gene bank of *Rhizobium meliloti. Proc Natl Acad Sci USA* **77**: 7347-7351.

Gallagher, L.A., Velazquez, E., Brook Peterson, S., Charity, J.C., Hsu, F., Radey, M.C. *et al.* (2021) Genome-wide protein-DNA interaction site mapping using a double strand DNA-specific cytosine deaminase. *bioRxiv*: 2021.2008.2001.454665.

Hanahan, D., and Meselson, M. (1983) Plasmid screening at high colony density. *Methods Enzymol* **100**: 333-342.

Kessler, B., de Lorenzo, V., and Timmis, K.N. (1992) A general system to integrate *lacZ* fusions into the chromosomes of gram-negative eubacteria: regulation of the *Pm* promoter of the TOL plasmid studied with all controlling elements in monocopy. *Mol Gen Genet* **233**: 293-301.

Manoil, C., and Beckwith, J. (1985) Tn*phoA*: a transposon probe for protein export signals. *Proc Natl Acad Sci USA* **82**: 8129-8133.

Martinez-Garcia, E., and de Lorenzo, V. (2011) Engineering multiple genomic deletions in Gram-negative bacteria: analysis of the multi-resistant antibiotic profile of *Pseudomonas putida* KT2440. *Environ Microbiol* **13**: 2702-2716.

Martinez-Garcia, E., Nikel, P.I., Aparicio, T., and de Lorenzo, V. (2014) Pseudomonas 2.0: genetic upgrading of *P. putida* KT2440 as an enhanced host for heterologous gene expression. *Microb Cell Fact* **13**: 159.

Silva-Rocha, R., Martinez-Garcia, E., Calles, B., Chavarria, M., Arce-Rodriguez, A., de Las Heras, A. et al. (2013) The Standard European Vector Architecture (SEVA): a coherent platform for the analysis and deployment of complex prokaryotic phenotypes. *Nucleic Acids Res* **41**: D666-675.

Wong, S.M., and Mekalanos, J.J. (2000) Genetic footprinting with mariner-based transposition in *Pseudomonas aeruginosa*. *Proc Natl Acad Sci USA* **97**: 10191-10196.
